# Editorial: Bioinformatics tools (and web server) for cancer biomarker development, volume II

**DOI:** 10.3389/fgene.2022.959159

**Published:** 2022-10-10

**Authors:** Longxiang Xie, Liuyang Wang, Wan Zhu, Jing Zhao, Xiangqian Guo

**Affiliations:** ^1^ Department of Predictive Medicine, Institute of Biomedical Informatics, Bioinformatics Center, Henan Provincial Engineering Center for Tumor Molecular Medicine, School of Basic Medical Sciences, Academy for Advanced Interdisciplinary Studies, Henan University, Kaifeng, China; ^2^ Department of Molecular Genetics and Microbiology, School of Medicine, Duke University, Durham, NC, United States; ^3^ Department of Anesthesia, Stanford University, Stanford, CA, United States; ^4^ Department of Pathophysiology, Chongqing Medical University, Chongqing, China

**Keywords:** bioinformatics, webserver, prognostic, biomarker, TCGA, GEO

Cancer is a major disease and a heavy burden to public health. With the rapid development of high-throughput (HTP) technologies, increasing multi-omics data have been produced to identify biomarkers to facilitate the risk assessment, early detection, prognosis, and treatment response prediction of tumors, and these biomarkers have helped to successfully decrease the mortality rate of certain types of cancer patients (Zou and Wang, 2019). In this Research Topic, we collected 23 articles representing recent advanced biomarkers studies for tumor diagnosis, prognosis, and treatment response.

Due to the wide application of next generation sequencing technologies in tumor research in recent decades, a few public omics data depositories, including The Cancer Genome Atlas (TCGA), Gene Expression Omnibus (GEO) and International Cancer Genome Consortium (ICGC), have been established and have aggregated a large number of clinical tumor tissue omics data and clinical data of tumor patients. These public databases provide reliable data support and additional opportunities for comprehensive biomarker identification (Tomczak et al., 2015; Ren et al., 2020). Song et al. investigated the TCGA and ICGC data of hepatocellular carcinoma (HCC), and performed the differential expression analysis and Cox regression analysis to identify core genes associated with HCC clinical outcomes, and a 6-autophagy-related-gene-pair (ARGP) prognostic signature was identified for overall survival (OS) of HCC patients. Based on the TCGA and GEO datasets, Shen et al. identified 108 differentially expressed genes, which were mainly involved in the proliferation and metastasis of head and neck squamous cell carcinoma (HNSCC). Furthermore, they constructed a novel multi-factor prognostic model of HNSCC and verified the reliability of the prognostic model in an additional 36 HNSCC patients.

In recent years, more new multi-omics databases were available and provided more opportunities for cancer diagnosis, treatments, and prevention, such as Chinese Glioma Genome Atlas (CGGA), which contains a wide range of data derived from whole-exome sequencing (WES), mRNA sequencing and microarray, DNA methylation microarray, and microRNA microarray analyses, as well as comprehensive clinical data (Zhao et al., 2021). Bai et al. evaluated m6A methylation regulatory genes and constructed a prognostic model of low-grade glioma (LGG) based on data of 495 LGGs from TCGA and 172 LGGs from the CGGA. This model contained 5 m6A-methylation- related genes and might classify LGGs into high- or low-risk subgroups. High-throughput technologies have been extended to characterize genomic status including but not limited to DNA methylation modification, genetic alteration, and gene expression regulation. Dong et al. explored the expression profiling data of CEMIP in different kinds of human cancers, and found that CEMIP was a prognostic and metastatic biomarker of breast cancer (BC). Their finding also uncovered that gene expression of CEMIP was mediated by TP53 mutation and DNA hypomethylation.

By integrating public accessible datasets, several powerful bioinformatics webservers/tools including KM plotter, GEPIA (Gene Expression Profiling Interactive Analysis), Oncomine, and TIMER (Tumor Immune Estimation Resource), have been developed to analyze the association or differential expression of genes with (in) distinct clinical factors in tumors (Li et al., 2017; Li et al., 2021). These online webservers/tools would help researchers to discover novel prognostic biomarkers which might be related to tumorigenesis or tumor malignant progression, and can provide more data basis for prognosis and therapeutic target identification of tumors. Based on a series of online tools, such as TIMER, Oncomine, and GEPIA, Chen et al. systematically analyzed the expression and prognostic value of PER1 in ovarian cancer (OV), and showed that low PER1 expression was related with poor prognosis of OV patients and concluded that PER1 may be a novel prognostic biomarker for OV. Zhang et al. used GEPIA to evaluate key co-expressed genes with C1ORF112 in LGGs, and performed gene ontology (GO) and Kyoto Gene and Genome Encyclopedia (KEGG) pathway analyses using the DAVID tool and identified a relationship between C1ORF112 expression and immune cell infiltration by TIMER. Their data suggested that C1ORF112 was closely related to the OS of patients with LGG, and was a prognostic biomarker of LGG. Liu et al. explored the relationship between inhibitors of apoptosis proteins and non-small cell lung cancer (NSCLC) progression through online bioinformatics tools, and found that BIRC1 is a potential biomarker associated with OS and BIRC5 is a potential diagnostic and staging biomarker of NSCLC patients. By using multiple bioinformatics analyses tools, Zhu et al. identified and verified that high expression of TUBA1C is related to a poor prognosis of LGG patients. Based on the TCGA dataset, Fan et al. identified DEPDC1B as a potential biomarker for diagnosis and prognosis of liver hepatocellular carcinoma (LIHC). In addition, Zhu et al. found that DNTTIP1 is a prognostic biomarker associated with overall survival (OS) and disease-free survival (DFS) in LIHC as well by using the TCGA dataset. In a review of the CPNE family, Tang et al. summarized the expression pattern and clinical roles of CPNE family members in cancer development. As membrane-bound proteins, CPNEs can mediate cell dedifferentiation and immune microenvironment organization of tumors. High expression of CPNE1 and CPNE3 could predict adverse prognosis. In contrast, CPNE5 might act as a positive indicator for esophageal squamous cell carcinoma (ESCC) and multiple myeloma (MM).

Although many single-gene biomarkers have been reported, multi-gene signature is more beneficial and meaningful for cancer prognosis. Accumulated public microarray data and RNA-seq data offer the opportunities to develop multi-gene signatures (Xie et al., 2020). Using the LASSO and Cox regression models, Zhao et al. established and validated a seven-gene signature (AFAP1L2, CAMK1D, LOXL2, PIK3CG, PLEKHG1, RARRES2, and SPP1) which might serve as a prognosis stratification tool to predict survival outcomes of advanced lung adenocarcinoma (LUAD) patients. He et al. explored bladder cancer (BLCA) transcriptome from two GEO datasets and TCGA, and performed the differential expression analysis and weighted gene co-expression network analysis (WGCNA) to identify core genes, finally a three hub gene signature (VSIG2, PPFIBP2, and DENND2D) related to invasion was constructed to divide BLCA patients into high-risk and low-risk groups. The high-risk group showed a higher mortality rate than the low-risk group using a Kaplan-Meier curve. Using a gastric cancer (GC) TCGA dataset, Liang et al. established a ten gene pyroptosis-related prognostic model which was able to divide GC patients into high-risk and low-risk groups, and this pyroptosis-related model was further verified in two independent GSE84437 and GSE66229 datasets. Using univariate and multivariate Cox regression algorithms, Han et al. constructed a prognostic model consisting of 14 inflammatory-related genes that might estimate outcomes for HNSCC patients by RT-PCR.

Cancer immunotherapies have shown great benefits for multiple types of tumor, including LUAD, PAAD and BLAC (Hayes, 2021; Zhao and Subramanian, 2021). Immune-related bioinformatics analysis would facilitate the improved treatment of tumor patients, and is expected to find new therapeutic targets. By analyzing TCGA and GEO BLCA datasets, Dong et al. constructed an immune-related eight-gene signature which was positively associated with immunotherapy response and prognosis of BLCA patients. By exploring immune-related genes (IRGs) obtained from the Immunology Database and Analysis Portal (ImmPort) database, Wang et al. constructed a prognostic model for BLCA patients and further validated this model by cross-validation. Mao et al. screened 446 significant immune-related genes in pancreatic adenocarcinoma and developed an immune-based prognostic model that was related to the status of the PAAD immune microenvironment. In order to investigate the effect of alternative splicing (AS) on the prognosis of LUAD, Song et al. integrated the prognostic AS genes and corresponding splicing factors to make a new prognostic signature with higher predictive ability than the mRNA signature.

Most cancer biomarker studies examine individual genetic variables, transcriptome alterations, and impaired protein function as distinct risk and prognostic factors. Increasing studies have demonstrated that the non-coding RNA (ncRNA) regulation network plays a significant role in cancer development (Anastasiadou et al., 2018). Xing et al. used Cox regression analysis to identify three immune-related lncRNAs (AC124067.4, LINC02604, and MIR4435-2HG), which were correlated with OS in COAD patients, and might play a role in anti-tumor immunotherapy. By analyzing 259 ferroptosis-associated genes from FerrDb, RNA-seq data and clinicopathological characteristics from TCGA, Zheng et al. established a novel prognostic signature including 10 ferroptosis-related lncRNAs, which was associated with the outcome of LUAD and tumor immune response.

Identification of high risk cancer patients is an important way to improve the clinical outcome and further facilitate understanding of the mechanisms of tumorigenesis and progression (Alifrangis et al., 2019). Based on crosstalk factorization, Liu et al. developed a new pathway activity score estimation method, and established a new prognostic classification system for colorectal cancer (CC), by which they separated the cancer patients in TCGA and three GEO datasets into aggressive (G2) and moderate (G1) subgroups, this prognostic system was shown to provide a complement to the current staging system. Using the same method, Liu et al. analyzed and classified breast cancer into G1 (moderate) and G2 (aggressive) subgroups with different OS risk. The survival outcome of the G1 subgroup was significantly better than the G2 subgroup. Most importantly, this BC risk classifier was validated in multiple BC datasets.

This Research Topic emphasises how prosperous public accessible profiling datasets and bioinformatics tools can be when widely used in oncology research. Although the tools/webservers presented here need to be improved, such as multi-omics network mapping and multi-gene signature assessment, these tools serve as a starting point for promoting the development of tumor diagnostic, prognostic, and prediction biomarker in the future.
